# The effect of different preventive strategies during total joint arthroplasty on periprosthetic joint infection: a network meta-analysis

**DOI:** 10.1186/s13018-024-04738-4

**Published:** 2024-06-18

**Authors:** Yongtao Wu, Xinni Xiang, Yimei Ma

**Affiliations:** 1 Department of Pediatrics, West China Second University Hospital, West China School of Medicine, Sichuan University, Chengdu, 610041 China; 2 Key Laboratory of Birth Defects and Related Diseases of Women and Children (Sichuan University), Ministry of Education, Chengdu, 610041 China; 3 West China School of Medicine, West China Hospital of Sichuan University, Chengdu, 610041 China

**Keywords:** Infection, Network meta-analysis, Total joint arthroplasty, Randomized controlled trial, Cohort study, Preventive strategy

## Abstract

**Background:**

Periprosthetic joint infection after total joint arthroplasty has a large incidence, and it may often require two or more stages of revision, placing an additional burden on clinicians and patients. The purpose of this network meta-analysis is to evaluate the effect of four different preventive strategies during total joint arthroplasty on the prevention of periprosthetic joint infection.

**Methods:**

The study protocol was registered at PROSPERO (CRD: 42,023,448,868), and the literature search databases included Web of Science, PubMed, OVID Cochrane Central Register of Controlled Trials, OVID EMBASE, and OVID MEDLINE (R) ALL that met the requirements. The network meta-analysis included randomized controlled trials, retrospective cohort studies and prospective cohort studies with the outcome of periprosthetic joint infection. The gemtc R package was applied to perform the network meta-analysis to evaluate the relative results of different preventive strategies.

**Results:**

This network meta-analysis study included a total of 38 articles with 4 preventive strategies and negative controls. No improvement was observed in antibiotic-loaded bone cement compared with negative controls. Chlorhexidine showed the highest probability of delivering the best preventive effect, and povidone iodine had the second highest probability. Although vancomycin ranked after chlorhexidine and povidone iodine, it still showed a significant difference compared with negative controls. In addition, the incidence after applying chlorhexidine was significantly lower than that after applying negative controls and vancomycin. In the heterogeneity test between direct and indirect evidence, there was no apparent heterogeneity between them.

**Conclusion:**

The study indicated that chlorhexidine, povidone iodine and vancomycin showed significant efficacy in preventing periprosthetic joint infection after total joint arthroplasty, while antibiotic-loaded bone cement did not. Therefore, more high-quality randomized controlled trials are needed to verify the results above.

**Supplementary Information:**

The online version contains supplementary material available at 10.1186/s13018-024-04738-4.

## Introduction

In recent years, total joint arthroplasty (TJA) has been acknowledged as a common major orthopedic operation, which improves the quality of life and relieves the pain of patients [1, 2]. However, periprosthetic joint infection (PJI), also called surgical site infection (SSI) after TJA in some researches, is one of the major complications, affecting the survival rate of the prosthesis and increasing the burden of medical insurance [3, 4]. In the study by Bhaveen H et al. [1], PJI patients undergoing two-stage revision cost nearly five times as much, nearly $100,000, compared to patients with uncomplicated primary TKA. They were also admitted to the hospital about three times as often as cases of primary TKA without complications. There is a rate of 1–3% to catch this severe complication after TJA [5]. Irrigation and debridement can be used for the early treatment of PJI patients, but this strategy may lead to persistent colonization of resistant pathogens and the risk of multiple microbial infections [6]. Moreover, studies by Fehring et al. [7], Koyonos et al. [8], Odum et al. [9], and Deirmengian et al. [10] all showed low irrigation and debridement success rates of only approximately 30–40%. Meanwhile, a study by Filippo Migliorini et al. [11] showed that PJI has as many as 47 pathogens, which poses an obstacle to preventing PJI. In patients with chronic PJI, a two-stage exchange procedure is considered to be the gold standard. It involves the removal of all material at the first stage, and then a spacer is introduced and systemic antibiotics are administered to ensure that the patient is free of infection. Finally, the second stage is undertaken to introduce new components and recomplete the TJA [6]. A study by Corentin Pangaud et al. [12] analyzed 18 articles for a two-stage exchange procedure including 1086 patients. The eradication rate after two-stage surgery ranged from 54% to 100%, with an average rate of 84.8%, which means that patients may still suffer from PJI. However, those who were successfully eradicated were 8.8% more likely to become infected again. In addition, as the annual volume of TJA procedures is projected to rise, so will the rate of subsequent PJI. Therefore, finding effective preventive strategies for infection to reduce its occurrence is an urgent issue at present.

Many randomized controlled trials (RCTs) and cohort studies have been performed to explore preventive strategies to reduce the incidence rate of PJI after TJA, such as applying vancomycin powder [13], povidone iodine wash [14], antibiotic-loaded bone cement (ALBC) [15] and chlorhexidine wash [16]. However, there are some inconsistencies in their findings. For example, in the retrospective study by Nick et al. [17], vancomycin powder had a significant preventive effect on PJI. However, another study conducted by Ahmed et al. [4] indicated that the intervention group using vancomycin powder had no significant reduction in superficial infection rates. A study by Murray et al. [18] also noted that existing articles were not sufficiently persuasive in terms of efficacy to recommend routine use of topical vancomycin in total hip arthroplasty (THA) and total knee arthroplasty (TKA). Therefore, many researchers have already conducted systematic reviews and meta-analyses on the effects of different preventive strategies. A study performed by Zhi et al. [19] indicated that in both the THA and TKA groups, vancomycin powder treatment resulted in a significantly lower proportion of patients with PJI. In another study conducted by Naomi et al. [20], diluted povidone iodine lavage was significantly better than saline solution lavage for preventing PJI. In a study by Sujeesh et al. [21], preventive use of ALBC on average reduced the risk of primary TJA by 64% compared to the control group. As the classic meta-analysis can compare only two treatments, which strategy may provide a better efficacy to reduce the incidence rate of PJI after TJA is uncertain.

Network meta-analysis (NMA) is a valuable tool for the simultaneous comparison of more than two treatments [22]. This tool can be used to compare the effect of the four preventive strategies mentioned above and not using preventive strategies on the incidence rate of PJI. Therefore, this analysis was performed to (1) find a highly effective preventive method to reduce the incidence of PJI after TJA and provide a better choice for the clinic and (2) explore whether the four preventive strategies could produce statistically significant preventive effects and provide multiple options for clinical use.

## Materials and methods

### Search strategy

This meta-analysis was guided by the PRISMA guidelines (Preferred Reporting Items for Systematic Reviews and Meta-analysis), and the protocol was registered at PROSPERO (CRD42023448868). Web of Science, PubMed, OVID Cochrane Central Register of Controlled Trials, OVID EMBASE, and OVID MEDLINE (R) ALL were searched for related articles concerning the prevention of PJI after TJA. The commonly used four measures, namely, applying vancomycin powder, betadine wash, ALBC, and chlorhexidine wash, were included in this network meta-analysis. Articles published from the inception of these databases to 24th July 2023 were included. The following MeSH terms and their synonyms and abbreviations were used to find relevant studies: “Vancomycin,” “Chlorhexidine,” “Povidone-Iodine,” “ALBC,” “periprosthetic joint infection,” “arthroplasty,”, etc. The specific search strategy is shown in Supplementary File 1. Two authors independently screened the titles and abstracts of the retrieved articles to evaluate whether they should be included. If two authors disagreed, the final decision was discussed. The methodological quality of each included study was evaluated by using Cochrane ROB for RCTs and the Newcastle‒Ottawa Scale (NOS) for cohort studies. All comments were based on previously published studies. Thus, no ethical approval or patient consent is needed.

### Inclusion criteria and exclusion criteria

The inclusion criteria were based on the PICOS (participants, interventions, comparators, outcomes, and study design) approach [23]. The inclusion criteria were as follows: (1) The study population was patients without PJI when the study started. (2) There must be at least one measure of (Antibiotic-loaded bone cement, Vancomycin, Povidone iodine, Chlorhexidine). (3) Vancomycin should be used as powder. (4) Povidone iodine and chlorhexidine should be used for bathing. (5) There was no excess intervention compared to the control group, or there could be at least one measure of (Antibiotic-loaded bone cement, Vancomycin, Povidone Iodine, Chlorhexidine, normal saline, bone cement without antibiotic). (6) The outcome should include incidence of PJI with exact number or percentage. (7) The research type was a randomized controlled trial (RCT), retrospective cohort study or prospective cohort study. (8) The article should be published in English. The exclusion criteria included the following: (1) In addition to antibiotic-loaded bone cement, vancomycin, povidone iodine, and chlorhexidine, the experimental group had other interventions compared with the control group. (2) In the same experimental group, two antibiotics (antibiotic-loaded bone cement, vancomycin, povidone iodine, and chlorhexidine) were applied. (3) Interventions that had an impact on outcomes but were not present in the experimental group were introduced in the control group. (4) The research included subgroups, and the subgroups included at least one of the following: antibiotic-loaded bone cement, vancomycin, povidone iodine, and chlorhexidine. However, the exact incidence of PJI in the subgroups was not available. (5) The full text can’t be found. (6) It has not been peer reviewed.

### Data extraction and quality assessment

Two authors extracted relevant data from eligible articles independently. Extracted information was as follows: the last name of the first author, year of publication, study type, type of operation, treatments, study size, gender, age, body mass index (BMI) of the study population, and outcomes. If two authors disagreed, the final decision was discussed. The primary outcome of this study was the incidence of PJI. We expect to capture as many samples as possible in articles that meet the inclusion criteria to improve the reliability of the outcome. Therefore, as long as a patient is diagnosed with PJI after surgery, they will be included in the calculation of incidence, regardless of whether the follow-up time in the included studies is consistent.

### Statistical analysis

NMA was used to evaluate the efficacy of different preventive strategies on PJI. Bayesian NMA was performed in R software (version 3.6.2, R Foundation, Vienna, Austria) with the gemtc [24] package to compare direct and indirect therapies. In addition, the forest graph, ranking probability graph, and heterogeneity test between direct and indirect evidence were all assessed by R software with the gemtc package and then painted. In addition, the ranking probability line chart and the surface under the cumulative ranking curve (SUCRA) were also assessed and painted by the ggplot2 package [25]. To ensure convergence, the parameters of the Bayes iterations were set as n.adapt = 20,000 and n.iter = 50,000.

## Results

### Search results and study characteristics

**Fig. 1:**
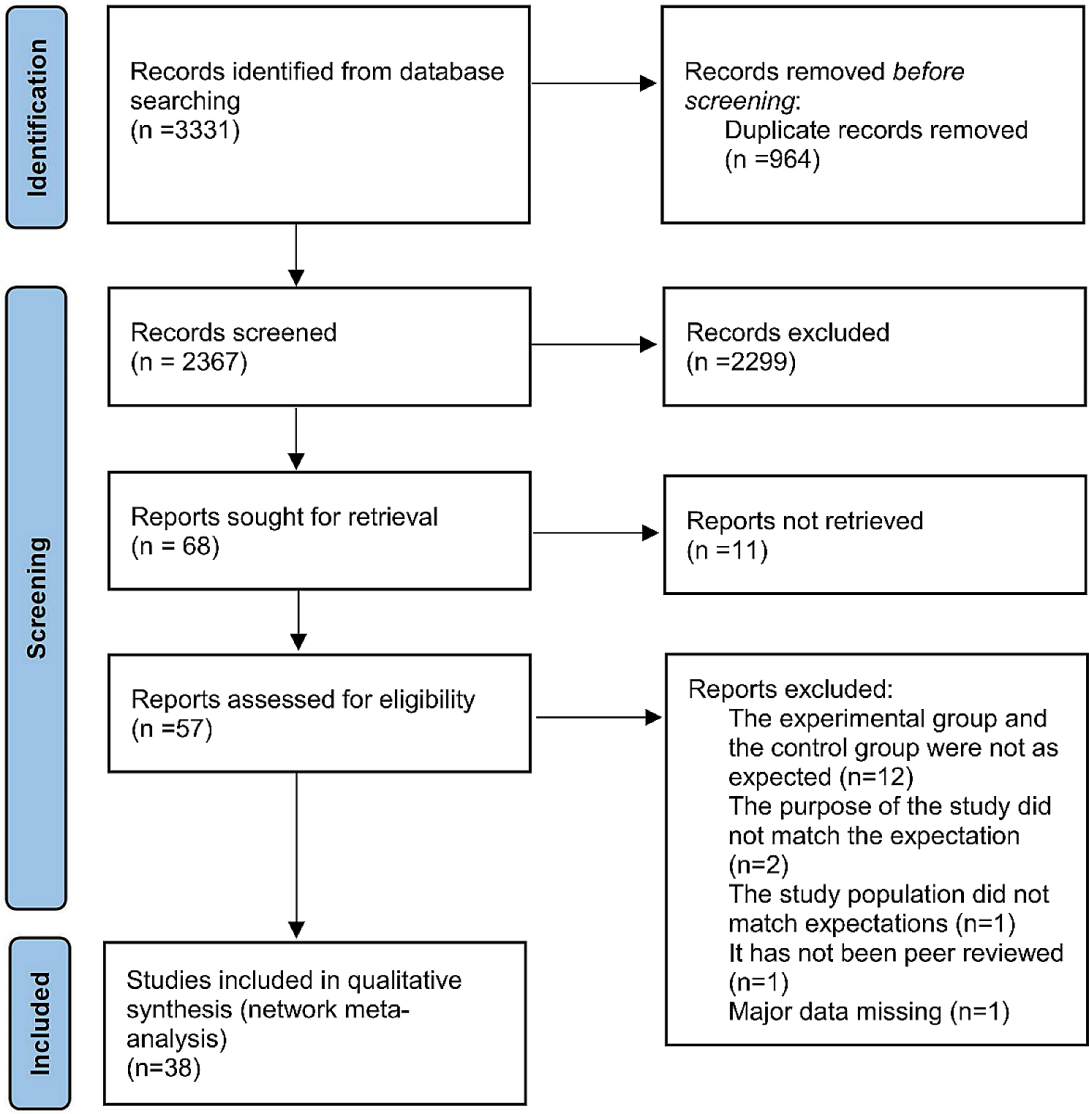
A flow chart showing the stages of retrieving articles and assessing the eligibility criteria for network meta-analysis of the effect of different preventive strategies on the prevention of PJI during total joint arthroplasty

A total of 3331 citations were involved in investigating the efficacy of 4 kinds of preventive strategies for PJI from five online databases (PubMed, OVID Cochrane Central Register of Controlled Trials, OVID EMBASE, OVID MEDLINE(R) ALL, Web of Science) by the predetermined search strategy. Finally, 38 studies were identified and considered eligible for the NMA. The publication years of the included articles ranged from 2008 to 2023. Among them, 8 articles compared ALBC with negative controls (NC) [26–33], 9 articles compared chlorhexidine bathing with NC [34–42], 4 articles compared povidone iodine lavage with NC [14, 43–45], and 14 articles compared vancomycin with NC [17, 46–58]. In addition, 2 articles compared chlorhexidine and povidone iodine bathing [16, 59], and 1 article compared povidone iodine bathing, vancomycin powder and NC [60]. The filtration process is shown in Fig. 1. In addition, the detailed characteristics of the 38 articles are shown in Supplementary File 2.

### Quality assessment

**Fig. 2:**
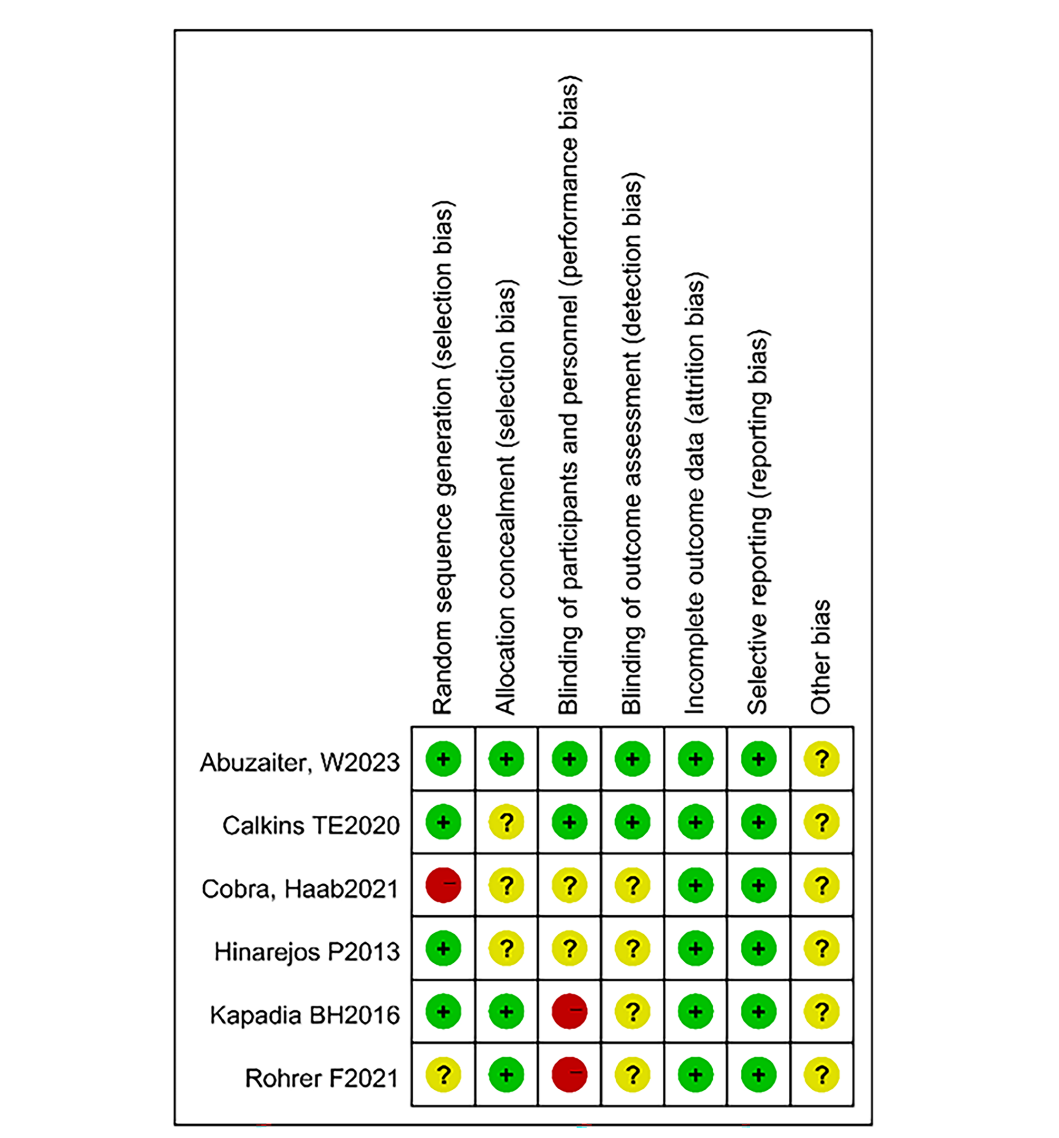
Risk of biases graph. Note: Red: high risk of bias. Green: low risk of bias. Yellow: uncertain risk of bias

Only 6 included studies were RCTs, among which 4 RCTs mentioned specific and highly reliable random sequence generation methods. Half of these RCTs did not provide clear allocation concealment, and only 2 RCTs used an effective method of blinding. All trials selected showed adequate and complete outcome data that met the inclusion criteria. All of these trials provided specific inclusion and exclusion criteria. The risk of biases graph is shown in Fig. 2.

Other 32 included studies were retrospective cohort studies or prospective cohort studies. The quality evaluation of these articles based on NOS will be presented in Supplementary File 3.

### Network meta-analysis results

#### NMA results

**Fig. 3:**
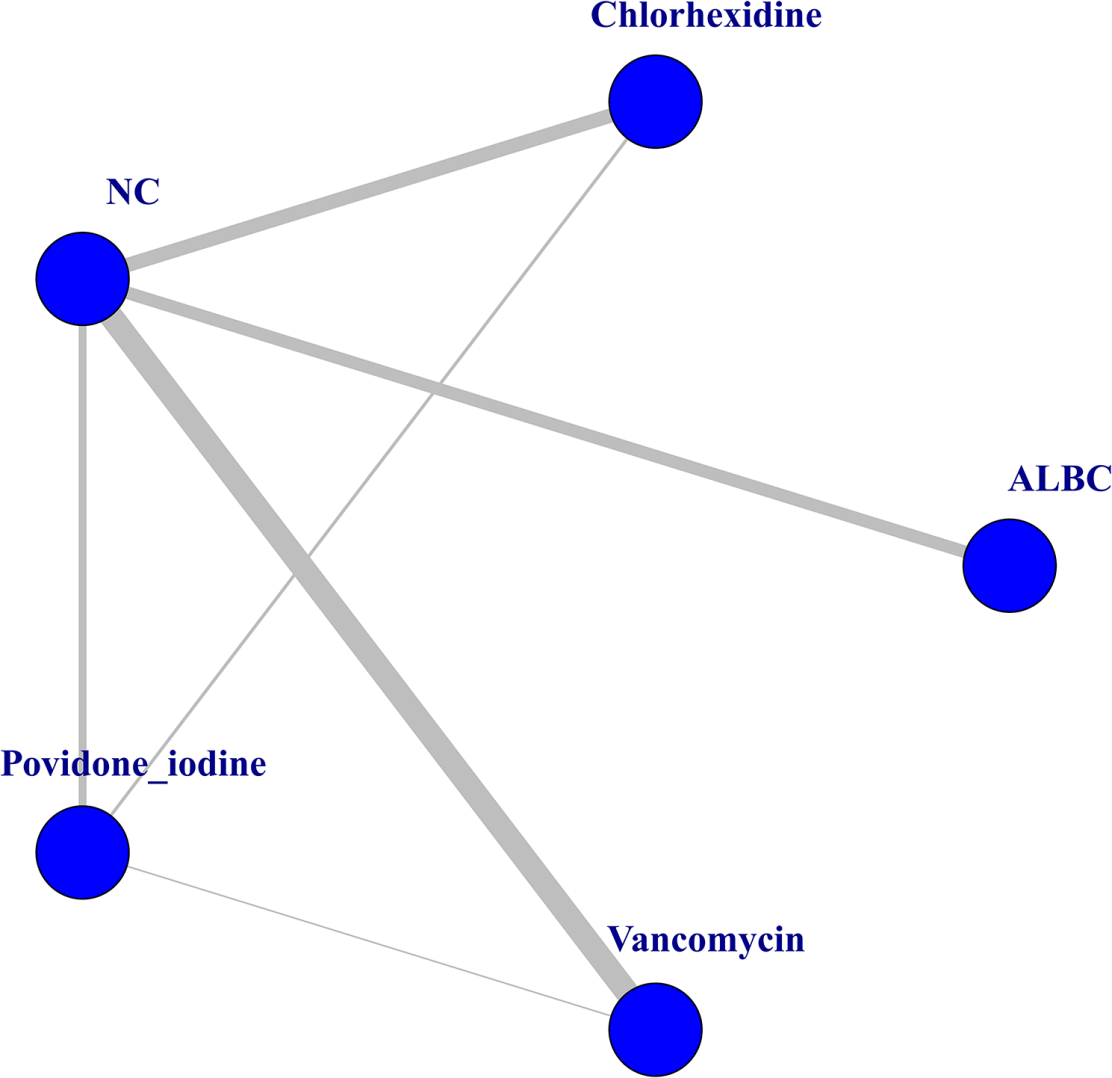
Network graph for the preventive strategies of PJI. Note: Each endpoint represents a kind of preventive strategy. Each line connecting 2 endpoints indicates that there were articles comparing 2 therapies. Line thickness depends on the number of trials that compared the two strategies. The graph shows the trials of PJI incidence after TJA. ALBC = Antibiotic-Loaded Bone Cement, NC = negative controls

A network graph of 5 kinds of preventive strategies was drawn with the help of R 3.6.2 software and a graphic package (Fig. 3). Apart from the four specific strategies (ALBC, chlorhexidine, povidone iodine, vancomycin), NC means that only normal saline, standard care, standard antibiotic application and bone cement without antibiotics were used in this group, and vancomycin powder, povidone iodine lavage, chlorhexidine lavage and ALBC were not used. The other operation parts were the same as those in the experimental group. Figure 3 details the indirect comparisons between different preventive strategies and direct comparisons between chlorhexidine and povidone iodine. In addition, the line thickness is proportional to the number of direct comparisons.

#### Forest plots

**Fig. 4:**
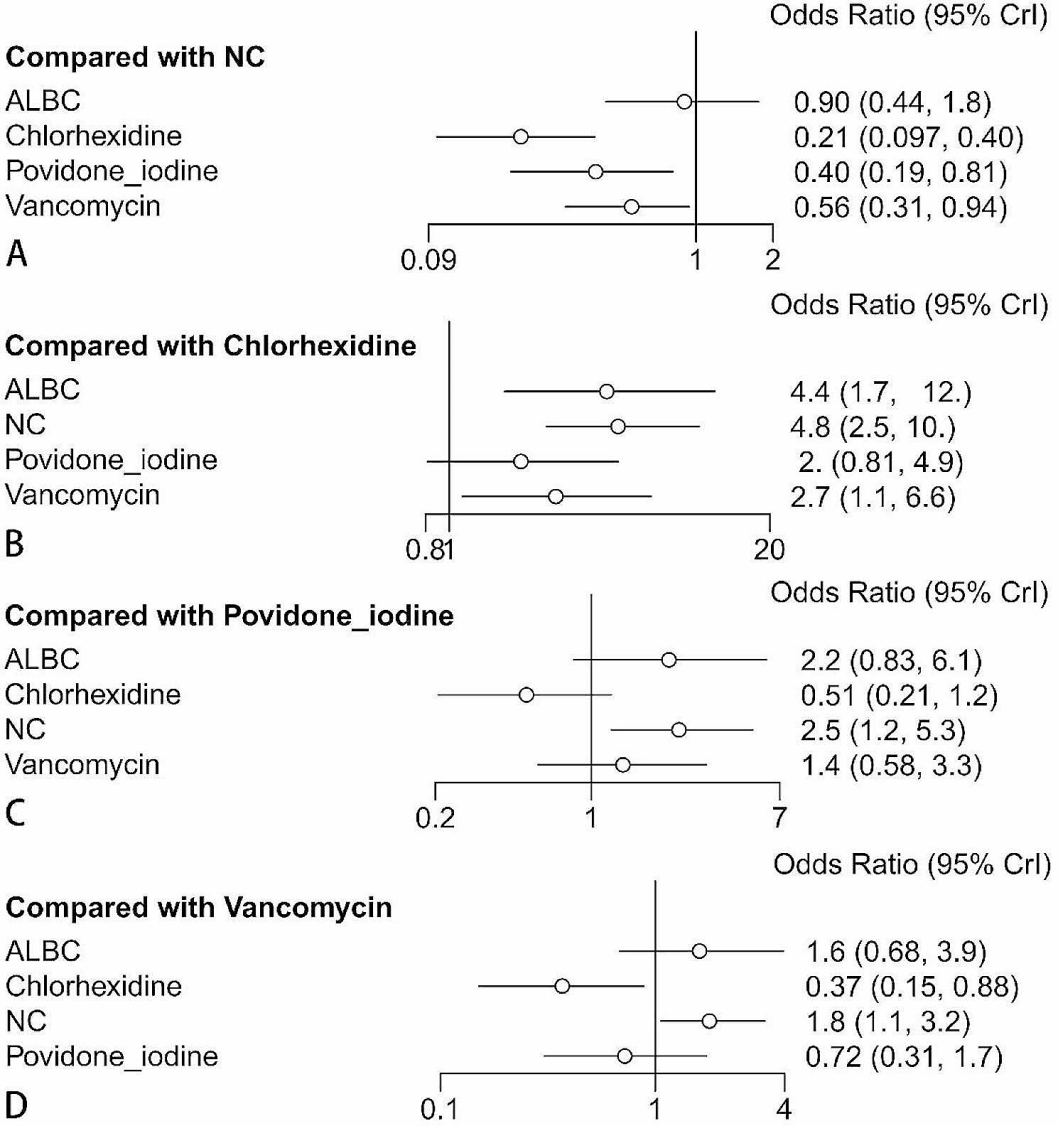
Forest graph on outcome. ALBC = antibiotic-loaded bone cement, NC = negative controls. (**A-D**): Forest plots (references were NC, chlorhexidine, povidone iodine, and vancomycin) indicate relative effect results compared with NC, chlorhexidine, povidone iodine, and vancomycin

The forest plots show the results of NMA on different preventive strategies. The ranking probabilities are shown in Fig. 4. The results of forest plots on PJI incidence of different preventive strategies (Fig. 4A) suggested that applying chlorhexidine, povidone iodine, and vancomycin showed a lower incidence of PJI than not using preventive strategies. (Compared with the NC, outcome: chlorhexidine OR = 0.21, 95% Crl [0.097, 0.40]; povidone iodine OR = 0.40, 95% Crl [0.19, 0.81]; vancomycin OR = 0.56, 95% Crl [0.31, 0.94]). The incidence of PJI in the patients using ALBC was not significantly lower than that in the control group. (Fig. 4A) (compared with NC, outcome: ALBC OR = 0.90, 95% Crl [0.44, 1.8].

In these three methods that showed a lower incidence of PJI than NC, applying chlorhexidine showed a lower PJI incidence than applying vancomycin. (Compared with chlorhexidine, outcome: vancomycin OR = 2.7, 95% Crl [1.1, 6.6]). However, there was no significant difference in the incidence of PJI between patients treated with vancomycin and those treated with povidone iodine or between patients treated with chlorhexidine and those treated with povidone iodine. (Figs. 4B, C, D) (compared with povidone iodine, outcome: chlorhexidine OR = 0.51, 95% Crl [0.21, 1.2]; vancomycin OR = 1.4, 95% Crl [0.58, 3.3]).

#### Ranking probability

**Fig. 5:**
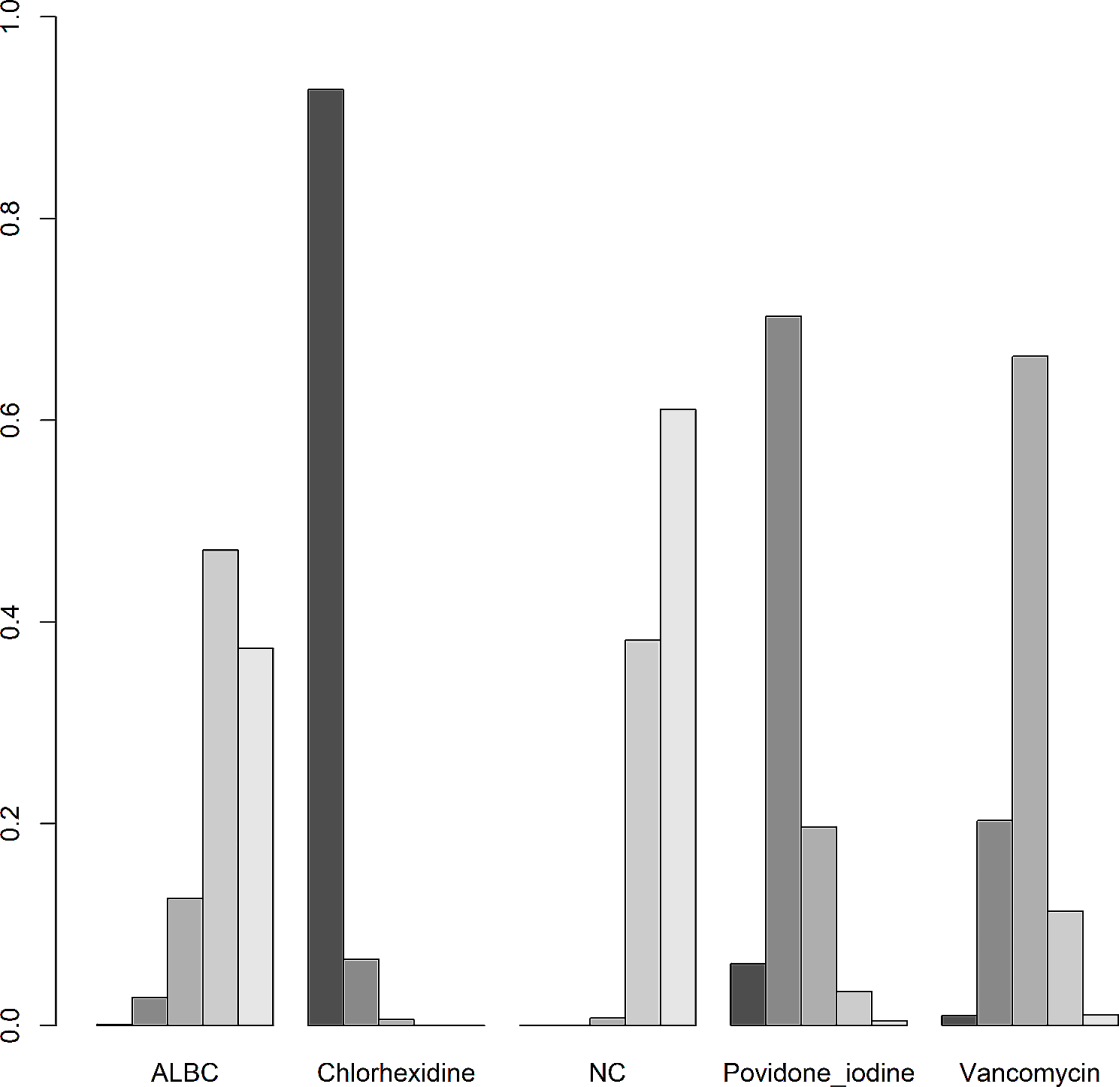
Ranking probability clustered plot of outcome. The heights of the bar columns represent the probability of being a certain rank. For every preventive strategy, bar columns from left to right represent ranks from best to worst

**Fig. 6:**
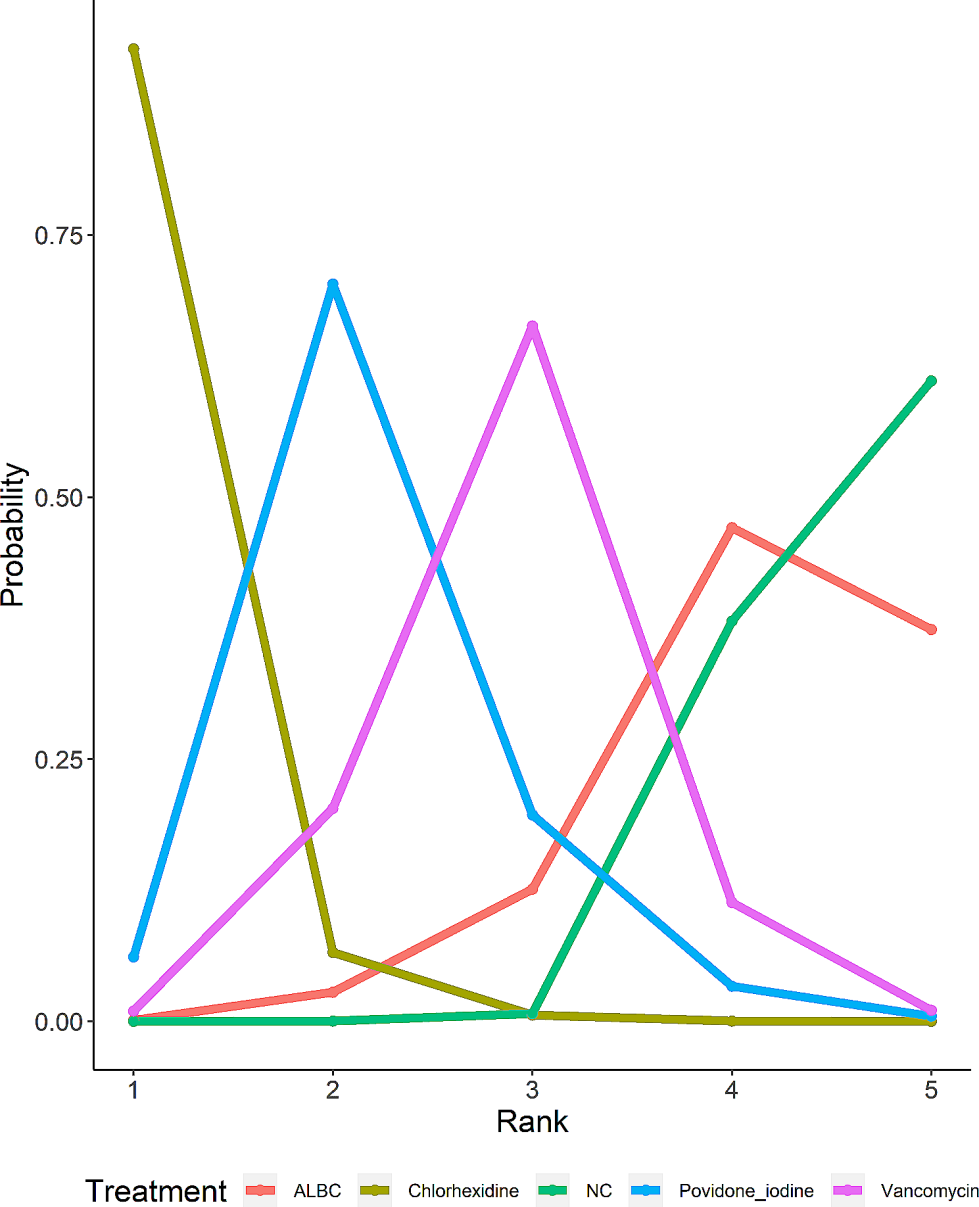
Ranking probability line chart of outcome. Each data point represents the probability of the preventive strategy to be a certain rank

**Fig. 7:**
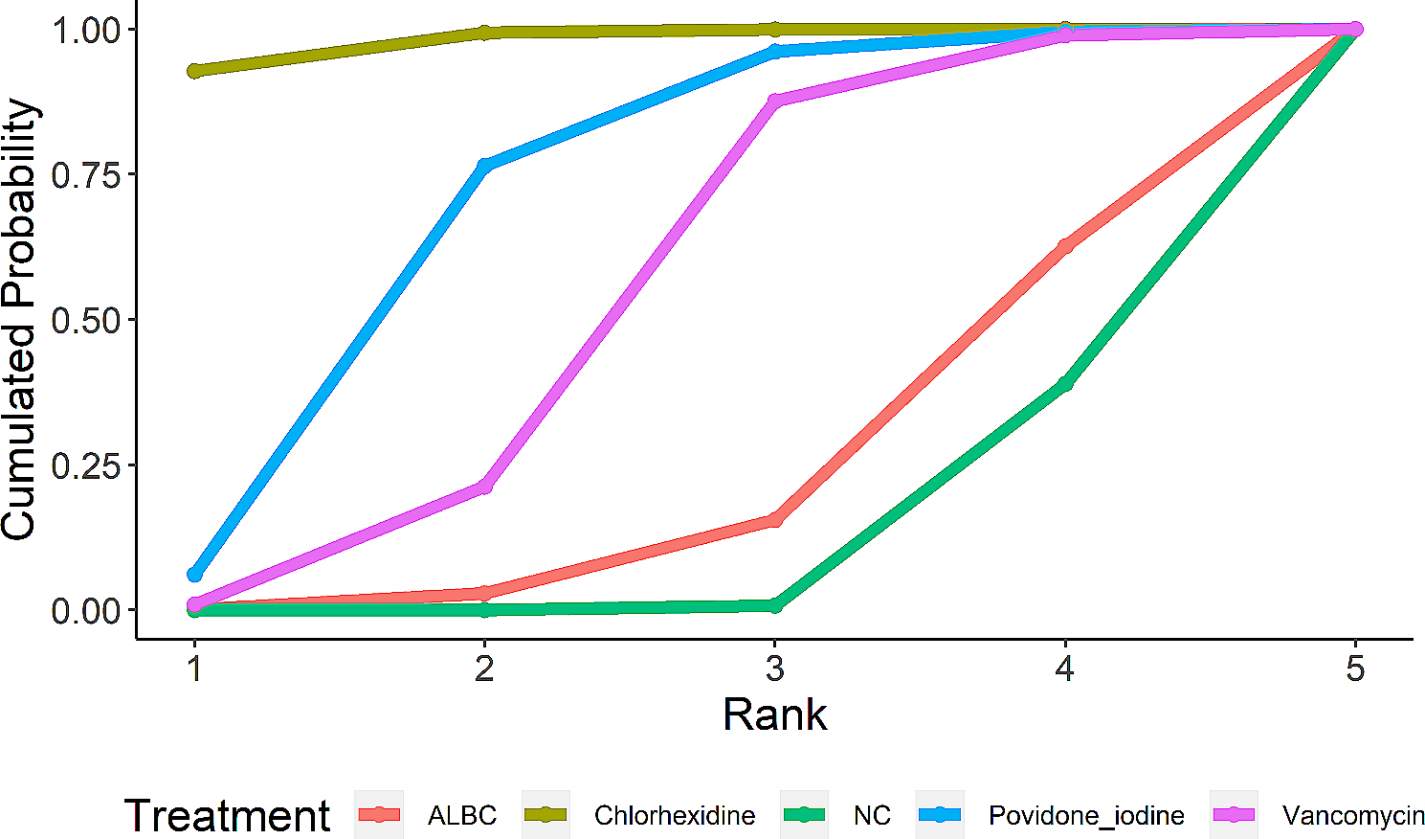
Ranking probability SUCRA graph of outcome. The larger the area under the curve, the more likely this preventive strategy is to be better

A clustered ranking plot and a ranking line chart were generated to present the NMA results visually. In addition, a SUCRA graph was also presented to show the results more specifically. The results are shown in Figs. 5, 6 and 7. The first two ranking plots were aimed at evaluating the highest probability of the best preventive strategy. With the outcome of incidence of PJI, the ranking plot showed that chlorhexidine had the most significant possibility of being the best strategy. Additionally, according to the SUCRA graph, chlorhexidine also had the largest area under the curve. All the graphs indicated that ALBC had the least probability, which was the same as NC.

#### Heterogeneity test

**Fig. 8:**
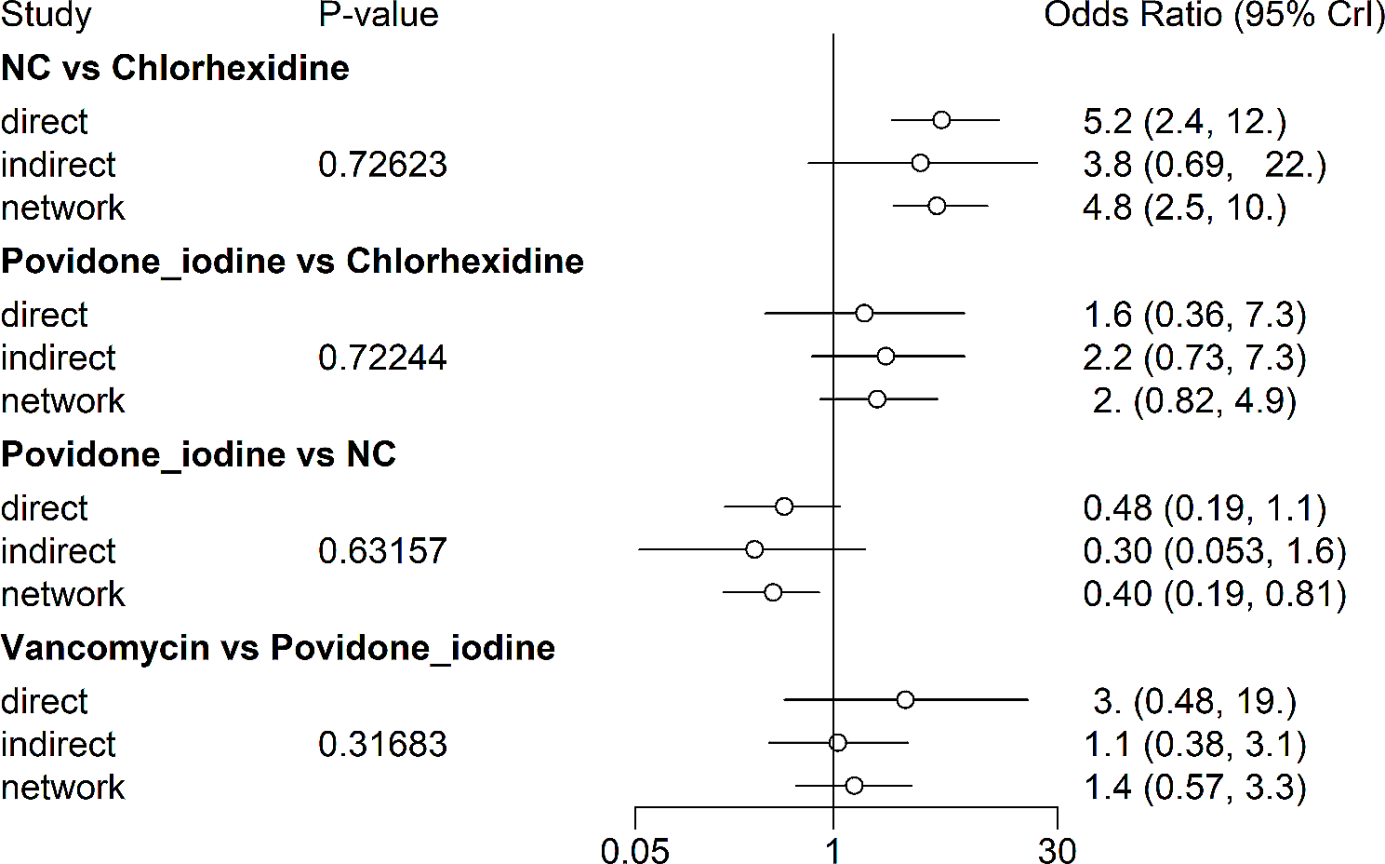
Heterogeneity test of outcomes. Note: NC = negative controls. *P* > 0.1 means that the heterogeneity is in an acceptable range

The node-splitting method [61] and its Bayesian *P* value were applied to assess the inconsistency between direct and indirect results (Fig. 8). The results showed *P* > 0.1, which indicated that the heterogeneity was in an acceptable range. According to the results, there was no significant heterogeneity in the outcomes. It was also believed that there was no significant heterogeneity in this network meta-analysis. However, most of the comparisons were indirect. Therefore, it calls for more trials that compare two or more preventive strategies directly.

#### Incidence comparison

**Fig. 9:**
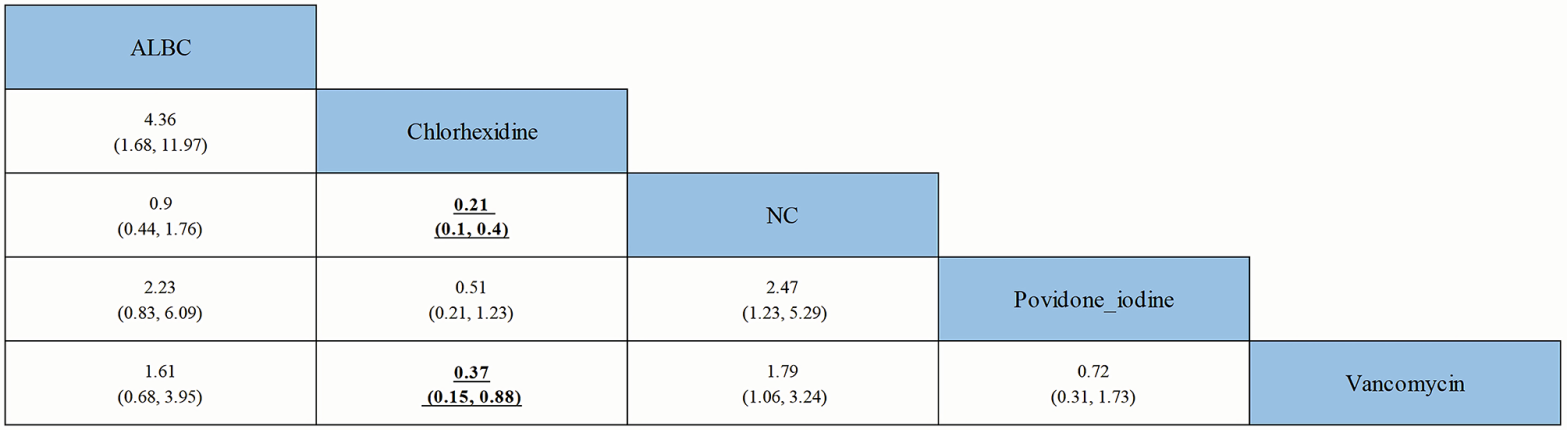
Comparison of the incidence of PJI by OR and 95% CrI among different preventive strategies. All results are displayed as the ratio of the Y axis versus the X axis. Bold font indicates a *p* value < 0.05

A comparison of the incidence of PJI by OR and 95% CrI among different preventive strategies is shown in Fig. 9. Compared with NC and vancomycin, chlorhexidine reduced the incidence of PJI (*p* value < 0.05). However, other comparisons did not show a significant superiority of one strategy over another.

## Discussion

PJI is an increasingly common inflammatory condition after TJA. Research by Andrew M. Schwartz shows that the number of revision THA operations increased by 36% from 2002 to 2014. According to their projections, the incidence of revision THA will even increase by 70% from 2014 to 2030. Moreover, for revision TKA, the revision rate will increase by a staggering 182% from 2014 to 2030 [62]. Therefore, it can be considered that the amount of revision surgery after the occurrence of PJI may increase to a certain degree. The study by Ferdinando et al. [63] mentioned quite a few preventive strategies, among which ALBC, chlorhexidine, povidone iodine and vancomycin were mentioned. However, there were some inconsistencies in regard to the efficacy of the four methods. It lacked comparisons between any two of the four methods. And there was no detailed evidence revealing that which of the strategies above could decrease the incidence of PJI most significantly. Therefore, it has practical significance to carry out an NMA to explore the efficacy of existing preventive strategies and determine the most effective method to provide some references for clinical work.

Vancomycin and ALBC have been widely used in clinical surgery to prevent PJI. However, a consensus only suggested that ALBC was effective while viewing vancomycin as not recommended [64]. Controversially, some studies have indicated that for decreasing the incidence of PJI, ALBC did not provide a significant difference, but vancomycin showed a significant difference [17, 26–29, 32, 48–51, 53, 54]. Moreover, a study indicated that ALBC increased the incidence of PJI [31]. In addition to vancomycin and ALBC, povidone iodine and chlorhexidine are also commonly used in TJA to prevent PJI. Povidone iodine wash and chlorhexidine wash are globally the most commonly used forms of infection prevention [65]. A consensus recommended chlorhexidine and povidone iodine as effective ways to reduce the incidence of PJI [66]. The evidence from the included studies also supported this opinion [4, 27–34]. Therefore, because of the controversy and the need to find the best preventive strategies, it is necessary to compare the effectiveness of these four preventive strategies. In this article, a total of five strategies, namely, ALBC, chlorhexidine, povidone iodine, vancomycin, and NC, were finally enrolled. Finally, the clinical outcome of the incidence of PJI was evaluated to directly indicate the efficacy of the four preventive strategies. In this network meta-analysis, the node-splitting method was performed to test the heterogeneity of indirect and direct evidence. It was obvious that *P* > 0.1, so the heterogeneity was in an acceptable range.

In previous studies, compared with NC, some studies showed lower PJI incidence after using vancomycin, ALBC, and povidone iodine [14, 17, 30, 33–41, 48–51, 53, 54, 60], but some showed opposite results [31, 44, 46, 56, 58] or no significant differences [26–29, 32, 42, 43, 45, 47, 52, 55, 57]. Most studies showed a lower risk of PJI incidence after using chlorhexidine. In this study, the results of traditional pairwise meta-analyses indicated that only three of the four strategies (chlorhexidine, povidone iodine and vancomycin) could significantly decrease the incidence of PJI compared with that of NC. However, the results did not support ALBC as a better strategy than NC for decreasing PJI incidence. The network meta-analysis results suggested that the rank probability for decreasing the incidence of PJI, from best to worst, was chlorhexidine, povidone iodine, vancomycin, ALBC, and NC, according to the ranking probability clustered plot and SUCRA probabilities.

The results were inconsistent with the consensus regarding the efficacy of vancomycin and ALBC in PJI prevention after TJA, which showed that compared with NC, vancomycin had a significant difference in the PJI incidence, while ALBC showed no significant difference. As the results were inconsistent with the consensus, more high-quality, large population RCTs should be conducted to better understand the effectiveness of these two strategies and to provide more valuable guidance for clinical practice. For chlorhexidine and povidone iodine, the results supported the consensus mentioned before because of the highest and second rank of chlorhexidine and povidone iodine in decreasing the incidence of PJI. However, only three RCTs on these two methods were included in this study, which may affect the reliability of the results to some extent, so it calls for more RCTs on the effectiveness of chlorhexidine and povidone iodine for PJI incidence. In addition, the comparisons of ALBC, chlorhexidine and vancomycin were indirect, which further proves that more high-quality randomized controlled trials with large samples and multiple subgroups are needed to verify the effectiveness of the four preventive strategies. Although there were direct results between chlorhexidine and povidone iodine and povidone iodine and vancomycin, only 3 studies were included, which was obviously insufficient.

To the best of our knowledge, this is the first network meta-analysis comparing the efficacy of four preventive strategies (ALBC, chlorhexidine, povidone iodine and vancomycin) and NC in preventing PJI, which shall provide better options and sufficient data support for clinical use. Moreover, direct comparisons of the five strategies were conducted, and indirect comparisons were performed by means of network meta-analysis to provide a hierarchy of these strategies. The study results further confirmed the effectiveness of chlorhexidine. Compared with a single RCT trial or a cohort study, the NMA results indicated a more integral conclusion. In a study conducted by Thomas et al. [67], disinfection rinsing with chlorhexidine solution during TJA reduced the risk of PJI in patients undergoing primary and revision total hip and knee replacement. In another meta-analysis of povidone iodine [20], a subgroup analysis of the saline control studies showed an odds ratio of 0.33 in the povidone iodine group, indicating a significant effect in preventing PJI. A study by Zhi et al. [19] also showed that topical application of vancomycin powder significantly reduced the incidence of PJI in primary TJA. For ALBC, the results from a meta-analysis performed by Yiqin et al. [68] did not show a significant difference in the incidence of PJI in patients who received and did not receive antibiotic-loaded cement. Similarly, a review that analyzed several different types of articles found that ALBC was not significantly effective in preventing PJI [69]. From the above existing research results, the NMA results can be verified with previous articles, providing more powerful and reliable evidence for the effects of the five strategies.

The limitations of this study are as follows: (1) few studies can be included; (2) most studies compared a preventive strategy with a negative control, but few studies compared two preventive strategies, so most of the comparisons were indirect; (3) the number of RCTs was not adequate, so the NMA included cohort studies, which means that the level of evidence in the original studies included may not have been high enough; (4) the results were merely analyzed in consideration of efficacy, without consideration of different doses, adverse effects and cost‒benefit analysis; and (5) due to the limitation of the meta-analysis, only limited data from previously published articles could be obtained and thus could not specify patients’ baseline characteristics and demographics.

## Conclusions

The study demonstrated that chlorhexidine lavage and povidone iodine lavage showed significant efficacy for PJI, while the efficacy of vancomycin was less significant. Chlorhexidine lavage provided the most significant reduction in the incidence of PJI and may be the preferred strategy for PJI prevention. In addition, the efficacy of ALBC was not significantly different from that of NC. Therefore, more extensive clinical trials are required to investigate whether ALBC provides an adequate advantage over normal bone cement without antibiotics.

### Electronic supplementary material

Below is the link to the electronic supplementary material.

**Supplementary Material 1**: The search strategy of English database

**Supplementary Material 2**: Characteristics of included studies

**Supplementary Material 3**: Quality evaluation

## Data Availability

No datasets were generated or analysed during the current study.

## References

[1] Sloan M, Premkumar A, Sheth NP (2018). Projected Volume of Primary Total Joint Arthroplasty in the U.S., 2014 to 2030. J Bone Joint Surg Am.

[2] Neuprez A, Neuprez AH, Kaux JF, Kurth W, Daniel C, Thirion T, Huskin JP, Gillet P, Bruyère O, Reginster JY (2020). Total joint replacement improves pain, functional quality of life, and health utilities in patients with late-stage knee and hip osteoarthritis for up to 5 years. Clin Rheumatol.

[3] Kapadia BH, McElroy MJ, Issa K, Johnson AJ, Bozic KJ, Mont MA (2014). The economic impact of periprosthetic infections following total knee arthroplasty at a specialized tertiary-care center. J Arthroplasty.

[4] Saidahmed A, Sarraj M, Ekhtiari S, Mundi R, Tushinski D, Wood TJ, Bhandari M (2021). Local antibiotics in primary hip and knee arthroplasty: a systematic review and meta-analysis. Eur J Orthop Surg Traumatology: Orthopedie Traumatologie.

[5] Akcaalan S, Ozaslan HI, Caglar C, Şimşek ME, Citak M, Akkaya M. Role of biomarkers in periprosthetic joint infections. Diagnostics (Basel Switzerland). 2022;12(12). 10.3390/diagnostics12122958 PMC9777153 36552965

[6] Gehrke T, Alijanipour P, Parvizi J (2015). The management of an infected total knee arthroplasty. Bone Joint J.

[7] Fehring TK, Odum SM, Berend KR, Jiranek WA, Parvizi J, Bozic KJ, Della Valle CJ, Gioe TJ (2013). Failure of irrigation and débridement for early postoperative periprosthetic infection. Clin Orthop Relat Res.

[8] Koyonos L, Zmistowski B, Della Valle CJ, Parvizi J (2011). Infection control rate of irrigation and débridement for periprosthetic joint infection. Clin Orthop Relat Res.

[9] Odum SM, Fehring TK, Lombardi AV, Zmistowski BM, Brown NM, Luna JT, Fehring KA, Hansen EN (2011). Irrigation and debridement for periprosthetic infections: does the organism matter?. J Arthroplasty.

[10] Deirmengian C, Greenbaum J, Stern J, Braffman M, Lotke PA, Booth RE, Lonner JH (2003). Open debridement of acute gram-positive infections after total knee arthroplasty. Clin Orthop Relat Res.

[11] Migliorini F, Weber CD, Bell A, Betsch M, Maffulli N, Poth V, Hofmann UK, Hildebrand F, Driessen A (2023). Bacterial pathogens and in-hospital mortality in revision surgery for periprosthetic joint infection of the hip and knee: analysis of 346 patients. Eur J Med Res.

[12] Tarabichi S, Parvizi J (2023). Prevention of surgical site infection: a ten-step approach. Arthroplasty (London England).

[13] Otte JE, Politi JR, Chambers B, Smith CA (2017). Intrawound Vancomycin Powder Reduces Early Prosthetic Joint Infections in revision hip and knee arthroplasty. Surg Technol Int.

[14] Muwanis M, Barimani B, Luo L, Wang CK, Dimentberg R, Albers A (2023). Povidone-iodine irrigation reduces infection after total hip and knee arthroplasty. Arch Orthop Trauma Surg.

[15] Crego-Vita D, Aedo-Martín D, Garcia-Cañas R, Espigares-Correa A, Sánchez-Pérez C, Berberich CE (2022). Periprosthetic joint infections in femoral neck fracture patients treated with hemiarthroplasty - should we use antibiotic-loaded bone cement?. World J Orthop.

[16] Driesman A, Shen M, Feng JE, Waren D, Slover J, Bosco J, Schwarzkopf R (2020). Perioperative Chlorhexidine Gluconate Wash during Joint Arthroplasty has Equivalent Periprosthetic Joint infection rates in comparison to Betadine Wash. J Arthroplasty.

[17] Patel NN, Guild GN, Kumar AR (2018). Intrawound Vancomycin in primary hip and knee arthroplasty: a safe and cost-effective means to decrease early periprosthetic joint infection. Arthroplasty Today.

[18] Wong MT, Sridharan SS, Davison EM, Ng R, Desy NM (2021). Can Topical Vancomycin prevent Periprosthetic Joint infection in hip and knee arthroplasty? A systematic review. Clin Orthop Relat Res.

[19] Peng Z, Lin X, Kuang X, Teng Z, Lu S (2021). The application of topical Vancomycin powder for the prevention of surgical site infections in primary total hip and knee arthroplasty: a meta-analysis. Orthop Traumatol Surg Res.

[20] Kobayashi N, Kamono E, Maeda K, Misumi T, Yukizawa Y, Inaba Y (2021). Effectiveness of diluted povidone-iodine lavage for preventing periprosthetic joint infection: an updated systematic review and meta-analysis. J Orthop Surg Res.

[21] Sebastian S, Liu Y, Christensen R, Raina DB, Tägil M, Lidgren L (2020). Antibiotic containing bone cement in prevention of hip and knee prosthetic joint infections: a systematic review and meta-analysis. J Orthop Translation.

[22] Morsali M, Poorolajal J, Shahbazi F, Vahidinia A, Doosti-Irani A (2021). Diet therapeutics interventions for obesity: a systematic review and network Meta-analysis. J Res Health Sci.

[23] Hutton B, Salanti G, Caldwell DM, Chaimani A, Schmid CH, Cameron C, Ioannidis JP, Straus S, Thorlund K, Jansen JP, Mulrow C, Catalá-López F, Gøtzsche PC, Dickersin K, Boutron I, Altman DG, Moher D (2015). The PRISMA extension statement for reporting of systematic reviews incorporating network meta-analyses of health care interventions: checklist and explanations. Ann Intern Med.

[24] van Valkenhoef G, Lu G, de Brock B, Hillege H, Ades AE, Welton NJ (2012). Automating network meta-analysis. Res Synthesis Methods.

[25] Ito K, Murphy D (2013). Application of ggplot2 to Pharmacometric Graphics. CPT: Pharmacometrics Syst Pharmacol.

[26] Cieremans D, Muthusamy N, Singh V, Rozell JC, Aggarwal V, Schwarzkopf R (2023). Does antibiotic bone cement reduce infection rates in primary total knee arthroplasty?. Eur.

[27] Cobra H, Mozella AP, Labronici PJ, Cavalcanti AS, Guimaraes JAM (2021). Infection after primary total knee arthroplasty: a randomized controlled prospective study of the addition of antibiotics to bone cement. Rev.

[28] Hoskins T, Shah JK, Patel J, Mazzei C, Goyette D, Poletick E, Colella T 2nd, Wittig J. The cost-effectiveness of antibiotic-loaded bone cement versus plain bone cement following total and partial knee and hip arthroplasty. J. 2020;20:217–20. 10.1016/j.jor.2020.01.029 PMC7005330 32051672

[29] Anis HK, Sodhi N, Faour M, Klika AK, Mont MA, Barsoum WK, Higuera CA, Molloy RM (2019). Effect of Antibiotic-Impregnated Bone Cement in primary total knee arthroplasty. J Arthroplasty.

[30] Chan JJ, Robinson J, Poeran J, Huang HH, Moucha CS, Chen DD (2019). Antibiotic-loaded bone cement in primary total knee arthroplasty: utilization patterns and impact on complications using a National Database. J Arthroplasty.

[31] Gutowski CJ, Zmistowski BM, Clyde CT, Parvizi J (2014). The economics of using prophylactic antibiotic-loaded bone cement in total knee replacement. Bone Joint J.

[32] Hinarejos P, Guirro P, Leal J, Montserrat F, Pelfort X, Sorli ML, Horcajada JP, Puig L (2013). The use of erythromycin and colistin-loaded cement in total knee arthroplasty does not reduce the incidence of infection: a prospective randomized study in 3000 knees. J Bone Joint Surg Am.

[33] Nowinski RJ, Gillespie RJ, Shishani Y, Cohen B, Walch G, Gobezie R (2012). Antibiotic-loaded bone cement reduces deep infection rates for primary reverse total shoulder arthroplasty: a retrospective, cohort study of 501 shoulders. J Shoulder Elb Surg.

[34] Dai W, Fang F (2022). Use of Chlorhexidine-impregnated gauze for skin Preparation reduces the incidence of Peri-prosthetic Joint Infection after primary total knee arthroplasty: a prospective cohort with retrospective controls. Surg Infect (Larchmt).

[35] Kapadia BH, Zhou PL, Jauregui JJ, Mont MA (2016). Does Preadmission Cutaneous Chlorhexidine Preparation reduce Surgical Site infections after total knee arthroplasty?. Clin Orthop.

[36] Kapadia BH, Elmallah RK, Mont MA, Randomized A (2016). Clinical trial of Preadmission Chlorhexidine skin Preparation for Lower Extremity Total Joint Arthroplasty. J Arthroplasty.

[37] Kapadia BH, Jauregui JJ, Murray DP, Mont MA (2016). Does Preadmission Cutaneous Chlorhexidine Preparation reduce Surgical Site infections after total hip arthroplasty?. Clin Orthop.

[38] Kapadia BH, Johnson AJ, Daley JA, Issa K, Mont MA (2013). Pre-admission cutaneous chlorhexidine preparation reduces surgical site infections in total hip arthroplasty. J Arthroplasty.

[39] Rao N, Cannella B, Crossett LS, Yates AJ, McGough R (2008). 3rd, a preoperative decolonization protocol for staphylococcus aureus prevents orthopaedic infections. Clin Orthop.

[40] Pelfort X, Romero A, Brugues M, Garcia A, Gil S, Marron A (2019). Reduction of periprosthetic Staphylococcus aureus infection by preoperative screening and decolonization of nasal carriers undergoing total knee arthroplasty. Acta Orthop Traumatol Turc.

[41] Rao N, Cannella BA, Crossett LS, Yates AJ Jr., McGough RL 3rd, Hamilton CW. Preoperative screening/decolonization for Staphylococcus aureus to prevent orthopedic surgical site infection: prospective cohort study with 2-year follow-up. J Arthroplasty. 2011;26(8):1501–7. 10.1016/j.arth.2011.03.014 21507604

[42] Rohrer F, Wendt M, Noetzli H, Risch L, Bodmer T, Cottagnoud P, Hermann T, Limacher A, Gahl B, Bruegger J (2021). Preoperative decolonization and periprosthetic joint infections-A randomized controlled trial with 2-year follow-up. J Orthop Res.

[43] Calkins TE, Culvern C, Nam D, Gerlinger TL, Levine BR, Sporer SM (2020). Della Valle, dilute Betadine Lavage reduces the risk of Acute Postoperative Periprosthetic Joint infection in aseptic revision total knee and hip arthroplasty: a Randomized Controlled Trial. J Arthroplasty.

[44] Hart A, Hernandez NM, Abdel MP, Mabry TM, Hanssen AD, Perry KI (2019). Povidone-Iodine Wound Lavage to prevent infection after revision total hip and knee arthroplasty: an analysis of 2,884 cases. J Bone Joint Surg Am.

[45] Slullitel PA, Dobransky JS, Bali K, Poitras S, Bhullar RS, Kim PR (2020). Is there a role for Preclosure Dilute Betadine Irrigation in the Prevention of Postoperative Infection Following Total Joint Arthroplasty?. J Arthroplasty.

[46] Abuzaiter W, Bolton CA, Drakos A, Drakos P, Hallan A, Warchuk D, Woolfrey KGH, Woolfrey MR (2023). Is topical Vancomycin an option? A Randomized Controlled Trial to Determine the Safety of the topical use of Vancomycin Powder in preventing postoperative infections in total knee arthroplasty, as compared with Standard Postoperative Antibiotics. J Arthroplasty.

[47] Aljuhani WS, Alanazi AM, Alghafees MA, Sagor SH, Alhandi AA (2021). The efficacy of Vancomycin powder in total knee arthroplasty: a single-center study. Saudi Med J.

[48] Garofalo R, Fontanarosa A, De Giorgi S, Lassandro N, De Crescenzo A (2023). Vancomycin powder embedded in collagen sponge decreases the rate of prosthetic shoulder infection. J Shoulder Elb Surg.

[49] Khatri K, Bansal D, Singla R, Sri S (2017). Prophylactic intrawound application of Vancomycin in total knee arthroplasty. J Arthrosc Joint Surg.

[50] Matziolis G, Brodt S, Bohle S, Kirschberg J, Jacob B, Rohner E (2020). Intraarticular Vancomycin powder is effective in preventing infections following total hip and knee arthroplasty. Sci.

[51] Tahmasebi MN, Vaziri AS, Vosoughi F, Tahami M, Khalilizad M, Rabie H (2021). Low post-arthroplasty infection rate is possible in developing countries: long-term experience of local Vancomycin use in Iran. J Orthop Surg.

[52] Tan TL, Gomez MM, Kheir MM, Maltenfort MG, Chen AF (2017). Should Preoperative Antibiotics be tailored according to patient’s comorbidities and susceptibility to organisms?. J Arthroplasty.

[53] Winkler C, Dennison J, Wooldridge A, Larumbe E, Caroom C, Jenkins M, Brindley G. Do local antibiotics reduce periprosthetic joint infections? A retrospective review of 744 cases, Journal of Clinical Orthopaedics and Trauma. 2018;9(Supplement 1):S34–S39. 10.1016/j.jcot.2017.08.007 PMC5883907 29628696

[54] Xu X, Zhang X, Zhang Y, Chen C, Yu H, Xue E (2020). Role of intra-wound powdered Vancomycin in primary total knee arthroplasty. Orthop Traumatol Surg Res.

[55] Yavuz IA, Oken OF, Yildirim AO, Inci F, Ceyhan E, Gurhan U (2020). No effect of Vancomycin powder to prevent infection in primary total knee arthroplasty: a retrospective review of 976 cases. Knee Surg Sports Traumatol Arthrosc.

[56] Zastrow RK, Huang HH, Galatz LM, Saunders-Hao P, Poeran J, Moucha CS (2020). Characteristics of antibiotic Prophylaxis and Risk of Surgical Site infections in primary total hip and knee arthroplasty. J Arthroplasty.

[57] Honkanen M, Sirkeoja S, Karppelin M, Eskelinen A, Syrjanen J. Effect of non-cephalosporin antibiotic prophylaxis on the risk of periprosthetic joint infection after total joint replacement surgery: a retrospective study with a 1-year follow-up. Infect Prev Pract. 2023;5(2):(no pagination)(100285). 10.1016/j.infpip.2023.100285 PMC10200839 37223241

[58] Kheir MM, Tan TL, Azboy I, Tan DD, Parvizi J (2017). Vancomycin Prophylaxis for Total Joint Arthroplasty: incorrectly dosed and has a higher rate of periprosthetic infection Than Cefazolin. Clin Orthop.

[59] Lung BE, Le R, Callan K, McLellan M, Issagholian L, Yi J, McMaster WC, Yang S, So DH (2022). Chlorhexidine gluconate lavage during total joint arthroplasty may improve wound healing compared to dilute betadine. J Experimental Orthop.

[60] Shohat N, Goh GS, Harrer SL, Brown S (2022). Dilute povidone-iodine irrigation reduces the rate of Periprosthetic Joint infection following hip and knee arthroplasty: an analysis of 31,331 cases. J Arthroplasty.

[61] van Valkenhoef G, Dias S, Ades AE, Welton NJ (2016). Automated generation of node-splitting models for assessment of inconsistency in network meta-analysis. Res Synthesis Methods.

[62] Schwartz AM, Farley KX, Guild GN, Bradbury TL (2020). Projections and epidemiology of revision hip and knee arthroplasty in the United States to 2030. J Arthroplasty.

[63] Iannotti F, Prati P, Fidanza A, Iorio R, Ferretti A, Pèrez Prieto D, Kort N, Violante B, Pipino G, Schiavone Panni A, Hirschmann M, Mugnaini M, Francesco Indelli P. Prevention of periprosthetic joint infection (PJI): a clinical practice protocol in high-risk patients. Tropical Medicine and Infectious Disease. 2020;5(4). 10.3390/tropicalmed5040186 PMC7768381 33322463

[64] Parvizi J, Gehrke T, Chen AF (2013). Proceedings of the International Consensus on Periprosthetic Joint infection. Bone Joint J.

[65] Dobson PF, Reed MR (2020). Prevention of infection in primary THA and TKA. EFORT open Reviews.

[66] Tokarski AT, Blaha D, Mont MA, Sancheti P, Cardona L, Cotacio GL, Froimson M, Kapadia BH, Kuderna J, López JC, Matar WY, McCarthy J, Morgan-Jones R, Patzakis M, Schwarzkopf R, Shahcheraghi GH, Shang X, Virolainen P, Wongworawat MD, Yates A (2014). Perioperative skin preparation. J Arthroplasty.

[67] Wood T, Ekhtiari S, Mundi R, Citak M, Sancheti PK, Guerra-Farfan E, Schemitsch E, Bhandari M (2020). The Effect of Irrigation Fluid on Periprosthetic Joint Infection in total hip and knee arthroplasty: a systematic review and Meta-analysis. Cureus.

[68] Zhou Y, Li L, Zhou Q, Yuan S, Wu Y, Zhao H, Wu H (2015). Lack of efficacy of prophylactic application of antibiotic-loaded bone cement for prevention of infection in primary total knee arthroplasty: results of a meta-analysis. Surg Infect (Larchmt).

[69] Rodriguez-Merchan EC. Antibiotic-loaded bone cement in primary total knee arthroplasty: does it reduce the risk of periprosthetic joint infection? Hospital Practice. 1995/2020;48(4):188–195. 10.1080/21548331.2020.1769417 32459547

