# Supplementary material for: The effect of different preventive strategies during total joint arthroplasty on periprosthetic joint infection: a network meta-analysis
Source: J Orthop Surg Res. 2024 Jun 18;19:360. doi: 10.1186/s13018-024-04738-4 (PMC11184793; doi:10.1186/s13018-024-04738-4)
Supplement: Supplementary file 1 — Supplementary Material 1: The search strategy of English database [file 13018_2024_4738_MOESM1_ESM.docx]

**Supplementary File 1.** The search strategy of English database

| 1. **Pubmed** |
| --- |
| #1 randomized controlled trial [Title/Abstract]  #2 controlled clinical trial [Title/Abstract]  #3 retrospective [Title/Abstract]  #4 prospective [Title/Abstract]  #5 cohort [Title/Abstract]  #6 #1 OR #2 OR #3 OR #4 OR #5  #7 Vancomycin [MeSH Terms]  #8 Vancomycin Hydrochloride [Title/Abstract]  #9 Hydrochloride, Vancomycin [Title/Abstract]  #10 Vancomycin Sulfate [Title/Abstract]  #11 Sulfate, Vancomycin [Title/Abstract]  #12 Vancomycin-ratiopharm [Title/Abstract]  #13 Vancomycin Hexal [Title/Abstract]  #14 Vancomycine Dakota [Title/Abstract]  #15 AB-Vancomycin [Title/Abstract]  #16 Vanco Azupharma [Title/Abstract]  #17 Diatracin [Title/Abstract]  #18 Vanco-saar [Title/Abstract]  #19 Vancocin [Title/Abstract]  #20 Vancocin HCl [Title/Abstract]  #21 Vancomycin Lilly [Title/Abstract]  #22 Vancocine [Title/Abstract]  #23 Vancomicina Abbott [Title/Abstract]  #24 Vancomicina Chiesi [Title/Abstract]  #25 Vancomicina Combino Phar [Title/Abstract]  #26 Vancomicina Norman [Title/Abstract]  #27 Vancomycin Phosphate [Title/Abstract]  #28 Vancomycin Phosphate, Decahydrate [Title/Abstract]  #29 #6 OR #7 OR #8 OR #9 OR #10 OR #11 OR #12 OR #13 OR #14 OR #15 OR #16 OR #17 OR #18 OR #19 OR #20 OR #21 OR #22 OR #23 OR #24 OR #25 OR #26 OR #27 OR #28  #30 Chlorhexidine [MeSH Terms]  #31 Chlorhexidine Hydrochloride [Title/Abstract]  #32 Hydrochloride, Chlorhexidine [Title/Abstract]  #33 Tubulicid [Title/Abstract]  #34 Novalsan [Title/Abstract]  #35 Sebidin A [Title/Abstract]  #36 Chlorhexidine Acetate [Title/Abstract]  #37 Acetate, Chlorhexidine [Title/Abstract]  #38 MK-412A [Title/Abstract]  #39 MK 412A[Title/Abstract]  #40 MK412A [Title/Abstract]  #41 #30 OR #31 OR #32 OR #33 OR #34 OR #35 OR #36 OR #37 OR #38 OR #39 OR #40  #42 Povidone-Iodine [MeSH Terms]  #43 Povidone Iodine [Title/Abstract]  #44 Povidone-Iodines [Title/Abstract]  #45 PVP-I [Title/Abstract]  #46 PVP-Iodine [Title/Abstract]  #47 PVP Iodine [Title/Abstract]  #48 PVP-Iodines [Title/Abstract]  #49 Polyvinylpyrrolidone Iodine [Title/Abstract]  #50 Polyvinylpyrrolidone Iodines [Title/Abstract]  #51 Betadine [Title/Abstract]  #52 Betadines [Title/Abstract]  #53 Providine [Title/Abstract]  #54 Providines [Title/Abstract]  #55 Disadine [Title/Abstract]  #56 Disadines [Title/Abstract]  #57 Isodine [Title/Abstract]  #58 Isodines [Title/Abstract]  #59 Pharmadine [Title/Abstract]  #60 Pharmadines [Title/Abstract]  #61 Alphadine [Title/Abstract]  #62 Alphadines [Title/Abstract]  #63 Betaisodona [Title/Abstract]  #64 #42 OR #43 OR #44 OR #45 OR #46 OR #47 OR #48 OR #49 OR #50 OR #51 OR #52 OR #53 OR #54 OR #55 OR #56 OR #57 OR #58 OR #59 OR #60 OR #61 OR #62 OR #63  #65 ALBC [Title/Abstract]  #66 PMMA with antibiotic [Title/Abstract]  #67 bone cement [Title/Abstract]  #68 anti-infective [Title/Abstract]  #69 anti-bacterial [Title/Abstract]  #70 antibiotic cement [Title/Abstract]  #71 #65 OR #66 OR #67 OR #68 OR #69 OR #70  #72 #29 OR #41 OR #64 OR #71  #73 total hip arthroplasty [Title/Abstract]  #74 total knee arthroplasty [Title/Abstract]  #75 arthroplasty [Title/Abstract]  #76 periprosthetic joint infection [Title/Abstract]  #77 #73 OR #74 OR #75 OR #76  #78 #6 AND #72 AND #77 |
| 2. **OVID Cochrane Central Register of Controlled Trials/ OVID EMBASE/ OVID MEDLINE(R) ALL** |
| #1 randomized controlled trial.ab,kw,ti  #2 controlled clinical trial.ab,kw,ti  #3 retrospective.ab,kw,ti  #4 prospective.ab,kw,ti  #5 cohort.ab,kw,ti  #6 #1 OR #2 OR #3 OR #4 OR #5  #7 Vancomycin.sh  #8 Vancomycin Hydrochloride.ab,kw,ti  #9 Hydrochloride, Vancomycin.ab,kw,ti  #10 Vancomycin Sulfate.ab,kw,ti  #11 Sulfate, Vancomycin.ab,kw,ti  #12 Vancomycin-ratiopharm.ab,kw,ti  #13 Vancomycin Hexal.ab,kw,ti  #14 Vancomycine Dakota.ab,kw,ti  #15 AB-Vancomycin.ab,kw,ti  #16 Vanco Azupharma.ab,kw,ti  #17 Diatracin.ab,kw,ti  #18 Vanco-saar.ab,kw,ti  #19 Vancocin.ab,kw,ti  #20 Vancocin HCl.ab,kw,ti  #21 Vancomycin Lilly.ab,kw,ti  #22 Vancocine.ab,kw,ti  #23 Vancomicina Abbott.ab,kw,ti  #24 Vancomicina Chiesi.ab,kw,ti  #25 Vancomicina Combino Phar.ab,kw,ti  #26 Vancomicina Norman.ab,kw,ti  #27 Vancomycin Phosphate.ab,kw,ti  #28 Vancomycin Phosphate, Decahydrate.ab,kw,ti  #29 #6 OR #7 OR #8 OR #9 OR #10 OR #11 OR #12 OR #13 OR #14 OR #15 OR #16 OR #17 OR #18 OR #19 OR #20 OR #21 OR #22 OR #23 OR #24 OR #25 OR #26 OR #27 OR #28  #30 Chlorhexidine.sh  #31 Chlorhexidine Hydrochloride.ab,kw,ti  #32 Hydrochloride, Chlorhexidine.ab,kw,ti  #33 Tubulicid.ab,kw,ti  #34 Novalsan.ab,kw,ti  #35 Sebidin A.ab,kw,ti  #36 Chlorhexidine Acetate.ab,kw,ti  #37 Acetate, Chlorhexidine.ab,kw,ti  #38 MK-412A.ab,kw,ti  #39 MK 412A.ab,kw,ti  #40 MK412A.ab,kw,ti  #41 #30 OR #31 OR #32 OR #33 OR #34 OR #35 OR #36 OR #37 OR #38 OR #39 OR #40  #42 Povidone-Iodine.sh  #43 Povidone Iodine.ab,kw,ti  #44 Povidone-Iodines.ab,kw,ti  #45 PVP-I.ab,kw,ti  #46 PVP-Iodine.ab,kw,ti  #47 PVP Iodine.ab,kw,ti  #48 PVP-Iodines.ab,kw,ti  #49 Polyvinylpyrrolidone Iodine.ab,kw,ti  #50 Polyvinylpyrrolidone Iodines.ab,kw,ti  #51 Betadine.ab,kw,ti  #52 Betadines.ab,kw,ti  #53 Providine.ab,kw,ti  #54 Providines.ab,kw,ti  #55 Disadine.ab,kw,ti  #56 Disadines.ab,kw,ti  #57 Isodine.ab,kw,ti  #58 Isodines.ab,kw,ti  #59 Pharmadine.ab,kw,ti  #60 Pharmadines.ab,kw,ti  #61 Alphadine.ab,kw,ti  #62 Alphadines.ab,kw,ti  #63 Betaisodona.ab,kw,ti  #64 #42 OR #43 OR #44 OR #45 OR #46 OR #47 OR #48 OR #49 OR #50 OR #51 OR #52 OR #53 OR #54 OR #55 OR #56 OR #57 OR #58 OR #59 OR #60 OR #61 OR #62 OR #63  #65 ALBC.ab,kw,ti  #66 PMMA with antibiotic.ab,kw,ti  #67 bone cement.ab,kw,ti  #68 anti-infective.ab,kw,ti  #69 anti-bacterial.ab,kw,ti  #70 antibiotic cement.ab,kw,ti  #71 #65 OR #66 OR #67 OR #68 OR #69 OR #70  #72 #29 OR #41 OR #64 OR #71  #73 total hip arthroplasty.ab,kw,ti  #74 total knee arthroplasty.ab,kw,ti  #75 arthroplasty.ab,kw,ti  #76 periprosthetic joint infection.ab,kw,ti  #77 #73 OR #74 OR #75 OR #76  #78 #6 AND #72 AND #77 |
| 3. **WEB OF SCIENCE** |
| #1 andomized controlled trial/TS  #2 controlled clinical trial/TS  #3 retrospective/TS  #4 prospective/TS  #5 cohort/TS  #6 #1 OR #2 OR #3 OR #4 OR #5  #7 Vancomycin/TS  #8 Vancomycin Hydrochloride/TS  #9 Hydrochloride, Vancomycin/TS  #10 Vancomycin Sulfate/TS  #11 Sulfate, Vancomycin/TS  #12 Vancomycin-ratiopharm/TS  #13 Vancomycin Hexal/TS  #14 Vancomycine Dakota/TS  #15 AB-Vancomycin/TS  #16 Vanco Azupharma/TS  #17 Diatracin/TS  #18 Vanco-saar/TS  #19 Vancocin/TS  #20 Vancocin HCl/TS  #21 Vancomycin Lilly/TS  #22 Vancocine/TS  #23 Vancomicina Abbott/TS  #24 Vancomicina Chiesi/TS  #25 Vancomicina Combino Phar/TS  #26 Vancomicina Norman/TS  #27 Vancomycin Phosphate/TS  #28 Vancomycin Phosphate, Decahydrate/TS  #29 #6 OR #7 OR #8 OR #9 OR #10 OR #11 OR #12 OR #13 OR #14 OR #15 OR #16 OR #17 OR #18 OR #19 OR #20 OR #21 OR #22 OR #23 OR #24 OR #25 OR #26 OR #27 OR #28  #30 Chlorhexidine/TS  #31 Chlorhexidine Hydrochloride/TS  #32 Hydrochloride, Chlorhexidine/TS  #33 Tubulicid/TS  #34 Novalsan/TS  #35 Sebidin A/TS  #36 Chlorhexidine Acetate/TS  #37 Acetate, Chlorhexidine/TS  #38 MK-412A/TS  #39 MK 412A/TS  #40 MK412A/TS  #41 #30 OR #31 OR #32 OR #33 OR #34 OR #35 OR #36 OR #37 OR #38 OR #39 OR #40  #42 Povidone-Iodine/TS  #43 Povidone Iodine/TS  #44 Povidone-Iodines/TS  #45 PVP-I/TS  #46 PVP-Iodine/TS  #47 PVP Iodine/TS  #48 PVP-Iodines/TS  #49 Polyvinylpyrrolidone Iodine/TS  #50 Polyvinylpyrrolidone Iodines/TS  #51 Betadine/TS  #52 Betadines/TS  #53 Providine/TS  #54 Providines/TS  #55 Disadine/TS  #56 Disadines/TS  #57 Isodine/TS  #58 Isodines/TS  #59 Pharmadine/TS  #60 Pharmadines/TS  #61 Alphadine/TS  #62 Alphadines/TS  #63 Betaisodona/TS  #64 #42 OR #43 OR #44 OR #45 OR #46 OR #47 OR #48 OR #49 OR #50 OR #51 OR #52 OR #53 OR #54 OR #55 OR #56 OR #57 OR #58 OR #59 OR #60 OR #61 OR #62 OR #63  #65 ALBC/TS  #66 PMMA with antibiotic/TS  #67 bone cement/TS  #68 anti-infective/TS  #69 anti-bacterial/TS  #70 antibiotic cement/TS  #71 #65 OR #66 OR #67 OR #68 OR #69 OR #70  #72 #29 OR #41 OR #64 OR #71  #73 total hip arthroplasty/TS  #74 total knee arthroplasty/TS  #75 arthroplasty/TS  #76 periprosthetic joint infection/TS  #77 #73 OR #74 OR #75 OR #76  #78 #6 AND #72 AND #77 |
