# Supplementary material for: The effect of different preventive strategies during total joint arthroplasty on periprosthetic joint infection: a network meta-analysis
Source: J Orthop Surg Res. 2024 Jun 18;19:360. doi: 10.1186/s13018-024-04738-4 (PMC11184793; doi:10.1186/s13018-024-04738-4)
Supplement: Supplementary file 2 — Supplementary Material 2: Characteristics of included studies [file 13018_2024_4738_MOESM2_ESM.docx]

**Supplementary File 2.** Characteristics of included studies

| Study (first author) | Study Design | Year | Type of Operation | Treatment | Size | Gender(M/F) | Age(year) | BMI(kg/m2) | Incidence |
| --- | --- | --- | --- | --- | --- | --- | --- | --- | --- |
| Abuzaiter, W | RCT | 2023 | TKA | NC | 85 | 29/56 | 64 | 35.7 | 0 |
|  |  |  |  | topical VP | 80 | 33/47 | 66 | 33.4 | 3 |
| Aljuhani, W. S | RCS | 2021 | TKA | NC | 49 | 3/46 | NA | NA | 1 |
|  |  |  |  | topical VP | 49 | 13/36 | NA | NA | 0 |
| Garofalo, R | RCS | 2023 | TSA | NC | 405 | 116/289 | 71 | 27.3 | 13 |
|  |  |  |  | topical VP | 420 | 127/293 | 73 | 26.8 | 0 |
| Khatri, K | RCS | 2017 | TKA | NC | 64 | 32/19 | NA | NA | 8 |
|  |  |  |  | topical VP | 51 | 44/20 | NA | NA | 5 |
| Matziolis, G | RCS | 2020 | TJA | NC | 7863 | NA | NA | NA | 92 |
|  |  |  |  | topical VP | 1082 | NA | NA | NA | 4 |
| Patel, N. N | RCS | 2018 | TJA | NC | 112 | 48/64 | 64.9 | 31.1 | 3 |
|  |  |  |  | topical VP | 348 | 138/210 | 63.6 | 30.6 | 2 |
| Tahmasebi, M.N | RCS | 2021 | TKA | NC | 314 | 62/252 | 66.37 | NA | 42 |
|  |  |  |  | topical VP | 1710 | 317/1393 | 64.99 | NA | 39 |
| Tan, T. L | RCS | 2017 | TJA | NC | 5810 | 2024/3786 | 63.5 | 30.1 | 62 |
|  |  |  |  | topical VP | 2765 | 892/1873 | 64.6 | 30.3 | 36 |
| Winkler, C | RCS | 2018 | TKA | NC | 152 | 48/104 | 27 | NA | 12 |
|  |  |  |  | topical VP | 191 | 70/121 | 80 | NA | 6 |
|  |  |  | THA | NC | 97 | 47/50 | 60.4 | NA | 1 |
|  |  |  |  | topical VP | 133 | 61/72 | 58.8 | NA | 1 |
| Xu, X | RCS | 2020 | TKA | NC | 418 | 129/289 | 67.1 | 24.9 | 5 |
|  |  |  |  | topical VP | 437 | 121/316 | 66.9 | 25.3 | 0 |
| Yavuz, I. A | RCS | 2020 | TKA | NC | 502 | 154/348 | 63.4 | 28.9 | 5 |
|  |  |  |  | topical VP | 474 | 148/326 | 65.5 | 29 | 4 |
| Zastrow, R. K | RCS | 2020 | THA | NC | 323218 | 149619/173599 | 65 | NA | 623 |
|  |  |  |  | topical VP | 22370 | 10199/12171 | 65 | NA | 65 |
|  |  |  | TKA | NC | 615380 | 375040/240330 | 66 | NA | 1230 |
|  |  |  |  | topical VP | 50711 | 30937/19774 | 66 | NA | 150 |
| Honkanen, M | RCS | 2023 | TJA | NC | 26467 | 10612/15855 | 68 | 30 | 228 |
|  |  |  |  | topical VP | 72 | NA | NA | NA | 0 |
| Kheir, M. M | RCS | 2017 | TJA | NC | 5810 | NA | NA | NA | 62 |
|  |  |  |  | topical VP | 1828 | 1222/606 | NA | 30 | 32 |
| Cieremans D | RCS | 2023 | TKA | NC | 7980 | 2641/5339 | 65.88 | 32.09 | 63 |
|  |  |  |  | ALBC | 1386 | 403/983 | 65.81 | 33.4 | 7 |
| Cobra, Haab | RCT | 2021 | TKA | NC | 158 | 25/133 | 66 | 29.3 | 4 |
|  |  |  |  | ALBC | 128 | 28/100 | 68 | 30.4 | 2 |
| Hoskins T | RCS | 2020 | TKA and THA | NC | 3653 | 1653/2486 | 67.92 | 31.62 | 7 |
|  |  |  |  | ALBC | 486 |  |  |  | 3 |
| Anis HK | RCS | 2019 | TKA | NC | 8164 | 3068/5096 | 69 | 32 | 195 |
|  |  |  |  | ALBC | 4377 | 1653/2724 | 68 | 34 | 129 |
| Chan JJ | RCS | 2019 | TKA | NC | 861794 | 321341/540453 | 66 | NA | 3966 |
|  |  |  |  | ALBC | 322476 | 120682/201794 | 66 | NA | 1514 |
| Gutowski CJ | RCS? | 2014 | TKA | NC | 2390 | 817/1573 | 65.9 | NA | 23 |
|  |  |  |  | ALBC | 4060 | 1449/2611 | 65.7 | NA | 40 |
|  |  |  | THA | NC | 3054 | 1449/1605 | 62.4 | NA | 11 |
|  |  |  |  | ALBC | 4433 | 2194/2239 | 62.1 | NA | 25 |
| Hinarejos P | RCT | 2013 | TKA | NC | 1465 | 353/1112 | 76.06 | 31.74 | 38 |
|  |  |  |  | ALBC | 1483 | 346/1137 | 75.84 | 31.5 | 47 |
| Nowinski RJ | RCS | 2012 | RTSA | NC | 265 | 79/186 | 70 | NA | 8 |
|  |  |  |  | ALBC | 236 | 73/163 | 68 | NA | 0 |
| Dai W | PCS | 2022 | TKA | NC | 1033 | 446/587 | 66.9 | 26.1 | 44 |
|  |  |  |  | chlorhexidine gauze | 1218 | 961/1257 | 67.6 | 26.3 | 25 |
| Driesman A | RCS | 2020 | TKA and THA | NC | 1227 | 424/803 | 65.36 | 31.32 | 14 |
|  |  |  |  | chlorhexidine bath | 1159 | 439/720 | 64.92 | 30.9 | 9 |
| Kapadia BH | RCT | 2016 | TKA and THA | NC | 279 | 103/169 | 62 | 32.2 | 8 |
|  |  |  |  | chlorhexidine cloths | 275 | 96/171 | 61 | 31.4 | 1 |
| Kapadia BH | RCS | 2013 | THA | NC | 1901 | 836/1065 | 58 | 38 | 32 |
|  |  |  |  | chlorhexidine cloths | 557 | 235/322 | 56 | 29 | 3 |
| Kapadia BH | RCS | 2016 | THA | NC | 2846 | 1265/1581 | 59 | 29.9 | 46 |
|  |  |  |  | chlorhexidine cloths | 995 | 456/539 | 58.4 | 30.3 | 6 |
| Kapadia BH | RCS | 2016 | THA | NC | 2726 | 1060/1666 | 62 | 34 | 52 |
|  |  |  |  | chlorhexidine cloths | 991 | 379/612 | 62 | 34 | 3 |
| Rao N. | PCS | 2008 | TJA | NC | 345 | NA | NA | NA | 12 |
|  |  |  |  | chlorhexidine bath | 164 | NA | NA | NA | 0 |
| Pelfort X | RCS | 2019 | TKA | NC | 400 | 134/266 | 72.2 | NA | 17 |
|  |  |  |  | chlorhexidine soap | 403 | 125/278 | 72.4 | NA | 5 |
| Rao N | PCS | 2011 | TJA | NC | 571 | NA | NA | NA | 19 |
|  |  |  |  | chlorhexidine bath | 321 | NA | NA | NA | 0 |
| Rohrer F | RCT | 2021 | TJA | NC | 303 | 169/134 | 63 | 28 | 0 |
|  |  |  |  | chlorhexidine bath | 310 | 158/152 | 63 | 28 | 0 |
| Calkins TE | RCT | 2020 | TJA | NC | 234 | 89/145 | 64.8 | 32.8 | 8 |
|  |  |  |  | betadine lavage | 223 | 98/125 | 64.3 | 32.4 | 1 |
| Driesman A | RCS | 2020 | TJA | chlorhexidine wash | 1159 | 439/720 | 64.92 | 30.9 | 9 |
|  |  |  |  | betadine wash | 1227 | 424/803 | 65.36 | 31.32 | 14 |
| Hart A | RCS | 2019 | TKA | NC | 979 | 486/493 | 65.2 | 33.4 | 34 |
|  |  |  |  | betadine lavage | 503 | 224/279 | 65.8 | 33.4 | 21 |
|  |  |  | THA | NC | 1021 | 484/537 | 66.1 | 30.3 | 32 |
|  |  |  |  | betadine lavage | 381 | 201/180 | 67.7 | 30.9 | 12 |
| Lung BE | RCS | 2022 | TJA | chlorhexidine lavage | 204 | 92/112 | 67.3 | 30.6 | 3 |
|  |  |  |  | betadine lavage | 206 | 91/115 | 65.1 | 30 | 5 |
| Muwanis M | RCS | 2023 | TJA | NC | 1207 | 593/918 | 69.8 | NA | 45 |
|  |  |  |  | betadine lavage | 1511 | 476/731 | 71.8 | NA | 17 |
| Shohat N | RCS | 2022 | TJA | Vancomycin | 6805 | NA | NA | NA | 111 |
|  |  |  |  | betadine lavage | 8659 | NA | NA | NA | 48 |
|  |  |  |  | NC | 8659 | 3922/4737 | 63.4 | 30.4 | 139 |
|  |  |  |  | betadine lavage | 8659 | 3923/4736 | 63.3 | 30.5 | 52 |
| Slullitel PA | RCS | 2020 | TJA | NC | 5588 | 2433/3155 | 64.88 | 31.14 | 48 |
|  |  |  |  | betadine lavage | 2890 | 1389/1500 | 63.9 | 30.63 | 23 |

Note: ALBC: antibiotic-loaded bone cement; NA: not available; NC: saline, standard care, standard antibiotic application, common bone cement and etc. PCS: prospective cohort study; RCS: retrospective cohort study; RCT: randomized controlled trial; THA: Total hip arthroplasty; TJA: Total joint arthroplasty; TKA: Total knee arthroplasty; TSA: Total shoulder arthroplasty; VP: vancomycin powder
