# Supplementary material for: The effect of different preventive strategies during total joint arthroplasty on periprosthetic joint infection: a network meta-analysis
Source: J Orthop Surg Res. 2024 Jun 18;19:360. doi: 10.1186/s13018-024-04738-4 (PMC11184793; doi:10.1186/s13018-024-04738-4)
Supplement: Supplementary file 3 — Supplementary Material 3: Quality evaluation [file 13018_2024_4738_MOESM3_ESM.docx]

**Supplementary File 3.** Quality evaluation

| Study | Selection | | | | Comparability | Exposure | | | Quality Scores |
| --- | --- | --- | --- | --- | --- | --- | --- | --- | --- |
|  | Representativeness of the exposed cohort | Selection of the non -exposed cohort | Ascertainment of exposure | Demonstration that outcome of interest was not present at start of study | Comparability of cohorts on the basis of the design or analysis | Assessment of outcome | Was follow-up long enough for outcomes to occur | Adequacy of follow up of cohorts |  |
| Aljuhani, W. S 2021 | * | * | * | * |  | * | * | * | 7 |
| Garofalo, R 2023 | * | * | * | * | * | * | * | * | 8 |
| Khatri, K 2017 | * | * | * | * | ** | * | * | * | 9 |
| Matziolis, G 2020 | * | * | * | * |  | * | * | * | 7 |
| Patel, N. N 2018 | * | * | * | * | ** | * | * | * | 9 |
| Tahmasebi 2021 | * |  | * | * | * | * | * | * | 7 |
| Tan, T. L 2017 | * | * | * | * | * | * | * | * | 8 |
| Winkler, C 2018 | * |  | * | * | * | * | * | * | 7 |
| Xu, X 2020 | * |  | * | * | ** | * | * | * | 8 |
| Yavuz, I. A 2020 | * | * | * | * | ** | * | * | * | 9 |
| Zastrow, R. K 2020 | * | * | * | * |  | * |  | * | 6 |
| Honkanen, M 2023 | * | * | * | * |  | * | * | * | 7 |
| Kheir, M. M 2017 | * | * | * | * |  | * |  | * | 6 |
| Driesman, A. 2020 | * | * | * | * | ** | * | * | * | 9 |
| Hart, A 2019 | * | * | * | * | * | * | * | * | 8 |
| Lung, B. E 2022 | * | * | * | * | ** | * | * | * | 9 |
| Muwanis M 2023 | * |  | * | * | * | * | * | * | 7 |
| Shohat, N 2022 | * |  | * | * |  | * | * | * | 6 |
| Slullitel PA 2020 | * |  | * | * | * | * | * | * | 7 |
| Dai, W 2022 | * |  | * | * | ** | * | * | * | 8 |
| Kapadia, B. H 2016 | * | * | * | * | * | * | * | * | 8 |
| Kapadia, B. H 2013 | * | * | * | * | * | * | * | * | 8 |
| Kapadia, B. H 2016 | * | * | * | * | * | * | * | * | 8 |
| Rao, N 2008 | * | * | * | * |  | * | * | * | 7 |
| Pelfort X 219 | * |  | * | * | * | * | * | * | 7 |
| Rao, N 2011 | * | * | * | * |  | * | * | * | 7 |
| Cieremans, D 2023 | * | * | * | * |  | * |  | * | 6 |
| Hoskins, T 2020 | * | * | * | * |  | * |  | * | 6 |
| Anis, H. K 2019 | * | * | * | * |  | * | * | * | 7 |
| Chan, J. J 2019 | * | * | * | * | * | * |  | * | 7 |
| Gutowski 2014 | * |  | * | * |  | * | * | * | 6 |
| Nowinski RJ 2012 | * | * | * | * | ** | * | * | * | 9 |
